# Urinary and serum oxysterols in children: developmental pattern and potential biomarker for pediatric liver disease

**DOI:** 10.1038/s41598-020-63758-2

**Published:** 2020-04-21

**Authors:** Yugo Takaki, Tatsuki Mizuochi, Hajime Takei, Keisuke Eda, Ken-ichiro Konishi, Jun Ishihara, Masahiro Kinoshita, Naoki Hashizume, Yushiro Yamashita, Hiroshi Nittono, Akihiko Kimura

**Affiliations:** 1 Department of Pediatrics and Child Health, Kurume University School of Medicine, Kurume, Fukuoka Japan; 2 Junshin Clinic Bile Acid Institute, Meguro-ku, Tokyo Japan; 3 Department of Pediatric Surgery, Kurume University School of Medicine, Kurume, Fukuoka Japan

**Keywords:** Sterols, Mass spectrometry

## Abstract

Few reports describe oxysterols in healthy children or in children with liver disease. We aimed to determine whether developmental changes in urinary and serum oxysterols occur during childhood, and to assess whether oxysterols might be biomarkers for pediatric liver disease. Healthy children enrolled as subjects (36 and 35 for urine and serum analysis, respectively) included neonates, infants, preschoolers, and school-age children, studied along with 14 healthy adults and 8 children with liver disease. We quantitated 7 oxysterols including 4β-, 20(S)-, 22(S)-, 22(R)-, 24(S)-, 25-, and 27-hydroxycholesterol using liquid chromatography/electrospray ionization-tandem mass spectrometry. Urinary total oxysterols were significantly greater in neonates than in infants (*P* < *0.05*), preschoolers (*P* < *0.001*), school-age children (*P* < *0.001*), or adults (*P* < *0.001*), declining with age. Serum total oxysterols in neonates were significantly lower than in infants (*P* < *0.05*), preschoolers (*P* < *0.001*), school-age children (*P* < *0.05*), or adults (*P* < *0.01*). Compared with healthy children, total oxysterols and 24(S)-hydroxycholesterol in liver disease were significantly increased in both urine (*P* < *0.001* and *P* < *0.001*, respectively) and serum (*P* < *0.001* and *P* < *0.05*, respectively). Oxysterols in liver disease, particularly 24(S)-hydroxycholesterol, were greater in urine than serum. Oxysterols change developmentally and might serve as a biomarker for pediatric liver disease. To our knowledge, this is the first such report.

## Introduction

Oxysterols are 27-carbon derivatives of cholesterol produced by enzymatic reactions or free-radical oxidation^1^. They represent transport forms of cholesterol, such as 24(S)-hydroxycholesterol (24(S)-HC; cholest-5-en-3β,24S-diol), which is conveyed from brain to liver by the circulation. They also can bind to receptors within cell nuclei, such as liver X receptors. The varied biologic properties of oxysterols make them an informative subject for analysis in blood samples as biomarkers of oxidative stress, intermediates in bile acid biosynthesis, and messengers for cell signaling and cholesterol transport^2,3^. Pathogenic influences of oxysterols have been described in Alzheimer disease, carcinogenesis, cardiovascular disease, degenerative diseases, and liver diseases^4–7^. Although oxysterols have sometimes been thought to be little more than intermediates in cholesterol catabolism, many studies described their roles as regulators of lipid metabolism and inflammation.

Few reports have examined oxysterols in healthy children or in pediatric liver and neurologic disorders. Some recent reports advocate serum oxysterols as sensitive and specific biomarkers for Niemann-Pick disease type C^8,9^. However, little attention has been given to developmental patterns of oxysterols such as bile acids during childhood.

The present study aimed to determine whether predictable developmental changes in urinary and serum oxysterols occur during childhood, and to assess whether oxysterols might be biomarkers for pediatric liver disease.

## Results

Table 1 shows characteristics of subjects enrolled in this study. We collected 36 urine and 35 serum samples from healthy children 3 days to 15 years old, and 14 urine and 14 serum samples from adults 19 to 57 years. One subject was enrolled for urine samples obtained both as a neonate (age, 21 days) and as an infant (age, 3 months). Urine only was sampled in 15 children, serum only in 14, and both urine and serum in 21.

**Table 1:** Characteristics of subjects.

	Total	Neonates	Infants	Preschoolers	School-age	Adults
Urine						
N	50	10	8	8	10	14
Gender (M/F)	25/25	5/5	4/4	4/4	5/5	7/7
Age (median)	5.0 years	5.0 days	4.0 months	3.5 years	9.5 years	41.5 years
(range)	(3 days−57 years)	(3–25 days)	(1–10 months)	(2–5 years)	(7–15 years)	(19–57 years)
**Serum**						
N	49	6	7	14	8	14
Gender (M/F)	24/25	3/3	3/4	7/7	4/4	7/7
Age (median)	5.0 years	3.0 days	9.0 months	3.0 years	10.5 years	41.5 years
(range)	(3 days–57 years)	(3–5 days)	(3–11 months)	(1–5 years)	(7–14 years)	(19–57 years)

Neonates, under 1 month old; Infants, 1 to 11 months old; Preschoolers, 1 to 6 years old; School-age, 7 to 15 years old; Adults, over 18 years old; N, number of subjects; M, male; F, female.

**Figure 1:**
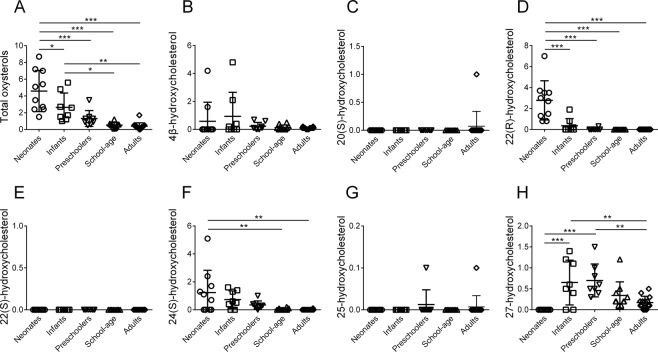
Urinary oxysterols in healthy children according to an enzymatic hydrolysis method. Horizontal lines represent means; vertical bars indicate standard deviations. Units are μmol/mol creatinine. (**A**) total oxysterols, (**B**) 4β-hydroxycholesterol, (**C**) 20(S)-hydroxycholesterol, (**D**) 22(R)-hydroxycholesterol, (**E**) 22(S)-hydroxycholesterol, (**F**) 24(S)-hydroxycholesterol, (**G**) 25-hydroxycholesterol, and (**H**) 27-hydroxycholesterol. Neonates, under 1 month after birth; Infants, 1 to 11 months old; Preschoolers, 1 to 6 years old; School-age, 7 to 15 years old; Adults, over 18 years old. *P < *0.05*; **P < 0.01; ***P < *0.001*.

Figure 1 shows urinary oxysterols in healthy neonates, infants, preschoolers, and school-age children, as well as healthy adults according to an enzymatic hydrolysis method. Urinary total oxysterols were significantly greater in neonates (mean ± standard deviation (SD), 4.61 ± 2.32 μmol/mol creatinine) than in infants (2.66 ± 1.60; *P* < *0.05*), preschoolers (1.31 ± 0.89; *P* < *0.001*), school-age children (0.52 ± 0.28; *P* < *0.001*), and adults (0.36 ± 0.38; *P* < *0.001*); in infants, urinary total oxysterols were significantly greater than in school-age children (*P* < *0.05*) and adults (*P* < *0.01*). Thus, urinary total oxysterols declined during development. Urinary 22(R)-hydroxycholesterol (22(R)-HC; cholest-5-en-3β,22R-diol), 24(S)-HC and 27-hydroxycholesterol (27-HC; 25(R)-cholest-5-en-3β,26-diol) showed significant differences among the age groups, while 22(R)-HC showed a developmental pattern resembling that of total oxysterols. Urinary 4β-hydroxycholesterol (4β-HC; cholest-5-en-3β,4β-diol), 20(S)-hydroxycholesterol (20(S)-HC; cholest-5-en-3β,20S-diol) and 25-hydroxycholesterol (25-HC; cholest-5-en-3β,25-diol) showed no significant differences, and 22(S)-hydroxycholesterol (22(S)-HC; cholest-5-en-3β,22S-diol) was not detected.

**Figure 2:**
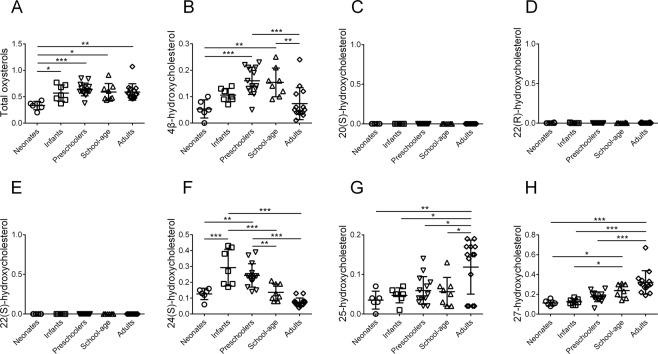
Serum oxysterols in healthy children according to an alkaline hydrolysis method. Horizontal lines represent means; vertical bars indicate standard deviations. Units are μmol/L. (**A**) total oxysterols, (**B**) 4β-hydroxycholesterol, (**C**) 20(S)-hydroxycholesterol, (**D**) 22(R)-hydroxycholesterol, (**E**) 22(S)-hydroxycholesterol, (**F**) 24(S)-hydroxycholesterol, (**G**) 25-hydroxycholesterol, and (**H**) 27-hydroxycholesterol. Neonates, under 1 month after birth; Infants, 1 to 11 months old; Preschoolers, 1 to 6 years old; School-age, 7 to 15 years old; Adults, over 18 years old. *P < *0.05*; **P < 0.01; ***P < *0.001*.

Figure 2 shows serum oxysterols in healthy children and adults according to an alkaline hydrolysis method. In neonates, serum total oxysterols (0.33 ± 0.07 μmol/L) were significantly lower than in infants (0.57 ± 0.14; *P* < *0.05*), preschoolers (0.64 ± 0.12; *P* < *0.001*), school-age children (0.59 ± 0.15; *P* < *0.05*, and adults (0.59 ± 0.15; *P* < *0.01*). Serum 4β-HC, 24(S)-HC, 25-HC, and 27-HC showed significant differences among the age groups, while serum 20(S)-HC, 22(R)-HC and 22(S)-HC were not detected.

Table 2 compares characteristics and serum laboratory results between normal controls (NC) and children with liver disease (LD) (n = 8 for each group). The LD group included 2 children with biliary atresia, 1 with congenital biliary dilatation, 3 with acute liver failure of unknown etiology, 1 with neonatal intrahepatic cholestasis caused by citrin deficiency, and 1 with autoimmune hepatitis. Their samples were collected at the time of diagnosis or before surgery. Serum alanine aminotransferase, γ-glutamyltransferase, total and direct bilirubin, and total bile acids were significantly greater in LD than in NC, while total cholesterol showed no significant difference.

**Table 2:** Characteristics and serum laboratory results in normal controls and children with liver disease.

	NC	LD	*P* value
N	8	8	
Gender (M/F)	5/3	5/3	*1.000*
Age, months (mean ± SD)	32 ± 21	15 ± 16	*0.124*
Alanine aminotransferase, U/L (mean ± SD)	21 ± 11	550 ± 623	*0.041*
γ-Glutamyltranspeptidase, U/L (mean ± SD)	13 ± 5	190 ± 143	*0.005*
Total bilirubin, mg/dL (mean ± SD)	0.44 ± 0.11	7.73 ± 4.20	*<0.001*
Direct bilirubin, mg/dL (mean ± SD)	0.05 ± 0.00	4.91 ± 3.03	*<0.001*
Total bile acids, μmol/L (mean ± SD)	7 ± 8	180 ± 70*	*0.002*
Total cholesterol, mg/dL (mean ± SD)	155 ± 25	167 ± 73	*0.694*

NC, normal controls; LD, children with liver disease; N, number of subjects; M, male; F, female; SD, standard deviation;

*N = 6.

**Figure 3:**
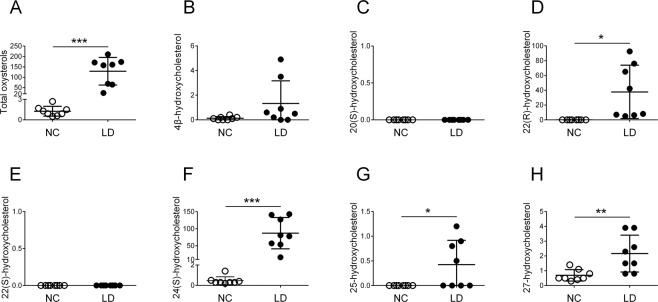
Urinary oxysterols in normal controls and children with liver disease according to an enzymatic hydrolysis method. Horizontal lines represent means; vertical bars are standard deviations. Units are μmol/mol creatinine. (**A**) total oxysterols, (**B**) 4β-hydroxycholesterol, (**C**) 20(S)-hydroxycholesterol, (**D**) 22(R)-hydroxycholesterol, (**E**) 22(S)-hydroxycholesterol, (**F**) 24(S)-hydroxycholesterol, (**G**) 25-hydroxycholesterol, and (**H**) 27-hydroxycholesterol. NC, normal controls; LD, children with liver disease. *P < *0.05*; **P < 0.01; ***P < *0.001*.

Figure 3 shows urinary oxysterols in NC and LD according to an enzymatic hydrolysis method. Urinary total oxysterols, 22(R)-HC, 24(S)-HC, 25-HC, and 27-HC were significantly greater in LD (respective means for NC vs. LD, 1.28 vs. 128.88 μmol/mol creatinine, *P* < *0.001*; 0.02 vs. 37.82, *P* < *0.05*; 0.46 vs. 87.13, *P* < *0.001*; 0 vs. 0.44, *P* < *0.05*; and 0.67 vs. 2.17 *P* < *0.01*). Urinary 4β-HC showed no significant difference, and neither 20(S)-HC nor 22(S)-HC was detected in either group. Urinary oxysterols in LD consisted mainly of 22(R)-HC and 24(S)-HC.

**Figure 4:**
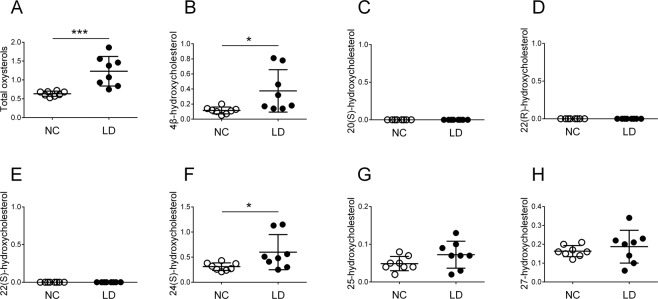
Serum oxysterols in normal controls and children with liver disease according to an alkaline hydrolysis method. Horizontal lines represent means; vertical bars are standard deviations. Units are μmol/L. (**A**) total oxysterols, (**B**) 4β-hydroxycholesterol, (**C**) 20(S)-hydroxycholesterol, (**D**) 22(R)-hydroxycholesterol, (**E**) 22(S)-hydroxycholesterol, (**F**) 24(S)-hydroxycholesterol, (**G**) 25-hydroxycholesterol, and (**H**) 27-hydroxycholesterol. NC, normal controls; LD, children with liver disease. *P < *0.05*; ***P < *0.001*.

Figure 4 shows serum oxysterols in NC and LD according to an alkaline hydrolysis method. Serum total oxysterols, 4β-HC, and 24(S)-HC were significantly greater in LD (respective means for NC vs. LD, 0.64 vs. 1.23 μmol/L, *P* < *0.001*; 0.11 vs. 0.38, *P* < *0.05*; and 0.31 vs. 0.60, *P* < *0.05*). Serum 25-HC and 27-HC showed no significant difference, and 20(S)-HC, 22(R)-HC, and 22(S)-HC were not detected in either group. Serum oxysterols in LD consisted mainly of 4β-HC and 24(S)-HC.

## Discussion

We examined urinary and serum oxysterols in healthy children and pediatric patients with liver disease, describing their developmental patterns in health, as well as assessing whether oxysterols might serve as a biomarker for pediatric liver diseases. Our data indicate that in healthy children, urinary total oxysterols in neonates are significantly higher than in any of the 4 older groups, and that infants had significantly higher values than school-age children and adults. Urinary total oxysterols showed a developmental decline. The opposite was true for serum total oxysterols; neonatal values were significantly lower than in the other 4 groups. Kimura *et al*. studied developmental patterns for urinary total bile acids in childhood and adulthood, finding that bile acids are significantly greater in neonates than in older subjects^10^. The 2 studies thus show a similarity between oxysterol metabolism and bile acid metabolism during the course of childhood.

In our study, the main urinary oxysterols during childhood were 4β-HC, 22(R)-HC, 24(S)-HC, and 27-HC. We found significant differences between age groups for 22(R)-HC, 24(S)-HC and 27-HC. During development 22(R)-HC sharply decreased, becoming undetectable beyond preschool. In contrast, 27-HC, the main urinary oxysterol in school-age children and adults was not detected in neonates. The main serum oxysterols were 4β-HC, 24(S)-HC, 25-HC, and 27-HC. We found significant differences among age groups for all 4 serum oxysterols. The predominant oxysterols differed between urine and serum samples. Serum 22(R)-HC was not detected in any age group, while 22(R)-HC was the main urinary oxysterol in neonates and infants. Furthermore, total oxysterols in neonates and infants were much more abundant in urine than serum. We suspect that oxysterols may have adverse biologic effects considering that they are cholesterol oxidation products, and therefore are excreted in the urine, as are unusual bile acids^11,12^.

In this study we also evaluated urinary and serum oxysterols in LD in comparison with age-matched NC. Among urinary oxysterols, 22(R)-HC, 24(S)-HC, 25-HC, and 27-HC were significantly increased in LD. In particular, 24(S)-HC, which accounts for most urinary total oxysterols in LD, showed the most significant elevations. Among serum oxysterols, on the other hand, 4β-HC and 24(S)-HC were significantly increased in LD. Based on these results, we believe that 24(S)-HC might serve as a biomarker for liver disease in children. We also performed a separate analysis of urinary and serum oxysterols in LD comparing cholestasis-derived samples (n = 4; serum direct bilirubin, >1.5 mg/dL) with acute liver failure samples (n = 3; including 2 patients with encephalopathy and 1 without). No significant differences were evident except for urinary 4β-HC (Supplementary Figure). To conclusively prove urinary and serum oxysterols to be a useful biomarker for pediatric liver disease, further study will be needed, examining larger numbers of subjects and including a variety of liver diseases such as cholestatic and non-cholestatic liver disease and acute liver failure with or without encephalopathy.

Synthesized mostly in the brain, 24(S)-HC has been suggested as a potential biomarker for neurologic diseases such as Alzheimer’s disease and multiple sclerosis^4,13^. As Meng *et al*. reported that serious cholestatic liver disease may increase formation and release of 24(S)-HC by the brain, urinary and serum 24(S)-HC may represent a prognostically useful parameter in clinically evaluating infants with severe cholestatic liver disease^14^. Considering our results in the light of these previous reports, we speculate that 24(S)-HC might possibly serve as a marker for brain damage in pediatric liver disease. Urinary 24(S)-HC might have a particular advantage because we detected a large amount of urinary 24(S)-HC in liver disease and urine is easier to collect from children than serum. Additional studies might examine oxysterols in cerebrospinal fluid, comparing patients with or without encephalopathy caused by acute liver failure.

Recently, Ikegami *et al*. reported increases in oxysterols such as 7α-HC, 25-HC, and 4β-HC in serum from adults with chronic hepatitis C virus infection^7^. As 4β-HC is synthesized in the liver by enzymes of the cytochrome P450 family such as CYP3A4^15^, we see a parallel with this previous report in our finding that 4β-HC was increased in children with liver disease.

When we analyzed 3 additional oxysterols–7α-hydroxycholesterol (cholest-5-en-3β,7α-diol), 7β-hydroxycholesterol (cholest-5-en-3β,7β-diol), and 7-oxo-cholesterol (cholest-5-en-3β-ol-7-one)–these showed a developmental pattern similar to the oxysterols (both urine and serum) such as 4β-HC, 24(S)-HC, and 27-HC in this study (data not shown). However, we excluded these oxysterols from our study because they seemed affected by oxidation, exceeding previously reported values in an inconsistent manner. We believe that sample storage over time at a temperature of −20 °C was responsible for these aberrations, since Helmschrodt *et al*. reported that these 3 oxysterols are susceptible to oxidation and increase during long-term storage at −20 °C^16^. Therefore, sample storage at −80 °C might be needed for these oxysterols. 25-HC can also be formed by oxidation on exposure to air; this may explain the high concentrations in Fig. 2.

Limitations of this study include a small number of subjects drawn from a single center and consideration of only a few kinds of liver disease. A prospective multicenter study such with a large number of subjects and including many types of liver disease including non-cholestatic and cholestatic conditions, as well as acute liver failure with and without encephalopathy will be needed.

## Conclusions

We characterized the developmental pattern of urinary and serum oxysterols during childhood and preliminarily explored use of oxysterols as a biomarker for pediatric liver disease. To our knowledge, this is the first report showing age specific changes of urinary and serum oxysterols in healthy children.

## Materials and Methods

### Study design and ethics

This research was designed as a prospective single-center observational study. Written informed consent was obtained from enrolled subjects’ parents and healthy adult volunteers. The study protocol complied with the ethical guidelines of the Declaration of Helsinki (2013 revision) and was approved by the Ethics Committee of Kurume University.

### Subjects and materials

Subjects included healthy children and LD who were under 16 years old and were clinically evaluated at Kurume University Hospital between February 2015 and August 2019, as well as healthy adult volunteers. Healthy individuals were divided into 5 age groups such as neonates (under 1 month after birth), infants (1 to 11 months old), preschoolers (1 to 6 years old), school-age children (7 to 15 years old), and adults (over 18 years old). NC to be compared with LD subjects were selected from the group of healthy children on the basis of ages and genders similar to those of individual children in the LD group, provided that both urine and serum were analyzed.

Urine and/or serum samples were collected from subjects after parental informed consent and stored under −20 °C before oxysterol analysis. Characteristics and serum laboratory results of subjects were obtained from medical records and clinical interviews.

### Oxysterol analysis by LC/ESI-MS/MS

In urine and serum, we quantitatively analyzed 10 oxysterols including 4β-HC, 7α-hydroxycholesterol, 7β-hydroxycholesterol, 7-oxo-cholesterol, 20(S)-HC, 22(S)-HC, 22(R)-HC, 24(S)-HC, 25-HC, and 27-HC by liquid chromatography/electrospray ionization-tandem mass spectrometry (LC/ESI-MS/MS). However, 3 oxysterols, specifically 7α-hydroxycholesterol, 7β-hydroxycholesterol and 7-oxo-cholesterol, showed excessive and unstable values when analyzed by our methods, probably reflecting storage time and temperature preceding sample preparation^16^. Therefore, this study included only the other 7 oxysterols. Urinary concentrations of individual oxysterols were corrected for creatinine concentration and expressed as micromoles per moles of creatinine.

### Sample preparation of oxysterols

For serum samples, 10 µL of d_6_-25-hydroxycholesterol (100 pmol/mL in methanol) as an internal standard and 10 μL of BHT (5 mg/mL in ethanol) were added to serum (10 μL). Alkaline hydrolysis was performed in 1 N ethanolic NaOH for 1 h at 37 °C. After addition of 0.5 mL of distilled water and 1 mL of hexane, the solution was vortex-agitated for approximately 2 min. The layer containing sterols was separated by centrifugation for 5 min at 1000 rpm. Sterols were extracted with 1 mL of hexane again, after which the hexane was allowed to evaporate and the residue was dissolved in 0.5 mL of hexane. Nonpolar sterols such as cholesterol were removed using aminopropyl SPE columns^17^ and taken up by an InertSep NH_2_ solid phase extraction cartridge (size, 100 mg/1 mL; GL Sciences, Tokyo, Japan). The column was rinsed with 1 mL of CHCl_3_/MeOH (1:1) and conditioned with 1 mL of hexane. The sample was transferred to the SPE column and was rinsed with 1 mL of hexane. Sterols then were eluted from the SPE column with 1 mL of CHCl_3_/MeOH (20:1), and solvents were allowed to evaporate. Derivatization to the nicotinyl ester was performed according to a previously reported method^18^ with minor modifications. Briefly, sterols were added to 100 μL of derivitization reagent (80 mg of nicotinic acid, 30 mg of N,N-dimethyl-4-aminopyridine, and 100 mg of 1-(3-dimethylaminopropyl)-3-ethylcarbodiimide hydrochloride in 1 mL of N,N-dimethylformamide). Samples were heated at 60 °C for 1 h, followed by addition of 1 mL of distilled water and 1 mL of hexane. After vortex-agitation of the mixture for approximately 2 min, nicotinyl ester derivatives were obtained by centrifugation for 5 min at 1000 rpm. The hexane layer was subjected to evaporation and reconstituted with 200 μL of acetonitrile. Aliquots of 10 μL were injected into the LC/ESI-MS/MS system.

For urine samples, enzymatic hydrolysis of oxysterol conjugates was performed according to a reported method^19^. To 200 μL of sample, 40 μl of reagent A and 10 μl of reagent B from the Glufatase set were added. The mixture was vortex-agitated for 10 s and allowed to stand for 5 min. After centrifugation for 5 min at 1500 rpm, 200 μL of supernatant was transferred to a 2-mL glass test tube along with 1 pmol of d_6_-25-hydroxycholesterol. Twenty microliters of acetate buffer from the Glufatase set was added, followed by 5 μL of glufatase enzyme from the Glufatase set and 5 μL of sulfatase type H-2. After incubation for 16 h at 37 °C, sterol extraction and derivatization were performed in as for serum samples.

Chemicals and reagents, preparation of standard solutions, and the LC/ESI-MS/MS analysis required for this oxysterol analysis are shown in Supplementary Methods.

### Enzyme specificity

Specificity of the enzyme hydrolysis for sulfated and glucuronidated oxysterols in urine was assessed based on previous reports^14,20^. Three milliliters of urine from a patient with cholestasis were purified using InertSep C18-B as follows. After pre-conditioning with 5 ml of methanol and 15 ml of H_2_O, the cartridge was washed with 4 ml of H_2_O and then eluted with 4 ml of 40% ethanol. The gel (PHP-GEL; Shimadzu Scientific Instruments, Kyoto, Japan) was equilibrated with 90% ethanol, washed with 2 ml of 0.1 N AcOH and eluted with 2 ml of ammonium acetate buffer at pH 6.3 in 90% ethanol. The solution was evaporated, re-dissolved in 200 μl of acetate buffer, and hydrolyzed either with or without the enzyme at 56 °C for 2 hr. After the hydrolyzed solution was purified using InertSep C18-B and evaporated, the residue was dissolved in 50 μl of water. Then 10 μl of solution was injected into the LC/ESI-MS/MS system. The peak on the chromatogram showing doubly conjugated oxysterol MS spectra (m/z 657; [M-H]^−^, m/z 577; [M-SO_3_-H]^−^, 481; [M-176-H]^−^) was compared between sample portions treated with and without the enzyme. The peak seen with sample unexposed to the enzyme was nearly eliminated in sample subjected to enzymatic hydrolysis.

### Laboratory tests

For subjects with serum samples, serum alanine aminotransferase, γ-glutamyltransferase, total and direct bilirubin, total bile acids, and total cholesterol were determined by routine methods.

### Statistical analysis

Continuous variables are expressed as the mean ± SD, and categorical variables as frequencies. ANOVA, Tukey-Kramer, Fisher’s exact, and Student *t* tests were used as appropriate. All statistical analysis was performed using GraphPad Prism software (version 6.05; GraphPad Software, San Diego, CA). Tests were two-sided. *P* values below 0.05 were considered to indicate statistical significance.

## Supplementary information

Supplementary Information.

## Data Availability

The data that support the findings of this study are available on request from the corresponding author.
