# Supplementary material for: Urinary and serum oxysterols in children: developmental pattern and potential biomarker for pediatric liver disease
Source: Sci Rep. 2020 Apr 21;10:6752. doi: 10.1038/s41598-020-63758-2 (PMC7174363; doi:10.1038/s41598-020-63758-2)

Urinary and serum oxysterols in children: developmental pattern and potential biomarker for pediatric liver disease

Yugo Takaki,[1] Tatsuki Mizuochi,[1,*] Hajime Takei,[2] Keisuke Eda,[1] Ken-ichiro Konishi,[1] Jun Ishihara,[1] Masahiro Kinoshita,[1] Naoki Hashizume,[3] Yushiro Yamashita,[1] Hiroshi Nittono,[2] and Akihiko Kimura[1]

[1]Department of Pediatrics and Child Health, Kurume University School of Medicine, Kurume, Fukuoka, Japan

[2]Junshin Clinic Bile Acid Institute, Meguro-ku, Tokyo, Japan

[3]Department of Pediatric Surgery, Kurume University School of Medicine, Kurume, Fukuoka, Japan

*** Corresponding author:** Tatsuki Mizuochi, MD, PhD, Department of Pediatrics and Child Health, Kurume University School of Medicine, 67 Asahi-machi, Kurume, Fukuoka 8300011, Japan.

Tel.: +81-942 317565; fax: +81-942 381792; email: mizuochi_tatsuki@kurume-u.ac.jp

Running title: Urinary and serum oxysterols in children

Supplementary Methods

Chemicals and reagents

4β-Hydroxycholesterol (cholest-5-en-3β,4β-diol), 7α-hydroxycholesterol (cholest-5-en-3β,7α-diol), 7-oxo-cholesterol (cholest-5-en-3β-ol-7-one), 20(S)-hydroxycholesterol (cholest-5-en-3β,20S-diol) 22(R)-hydroxycholesterol (cholest-5-en-3β,22R-diol) and 25-hydroxycholesterol (cholest-5-en-3β,25-diol) were purchased from Steraloids (Newport, RI, US). 22(S)-hydroxycholesterol (cholest-5-en-3β,22S-diol) was purchased from Sigma-Aldrich (St. Louis, MO, US). 24(S)-hydroxycholesterol (cholest-5-en-3β,24S-diol) was purchased Enzo Life Sciences (East Farmingdale, NY, US). 27-hydroxycholesterol (25(R)-cholest-5-en-3β,26-diol) was purchased from Research Plus (Farmingdale, NJ, US). d_6-25-Hydroxycholesterol (26,26,26,27,27,27-[$^2H_6$]25hydroxycholesterol) was purchased from Cayman Chemical (Ann Arbor, MI, US).

Nicotinic acid, N,N-dimethyl-4-aminopyridine (DMAP), N,N-dimethylformamide (DMF), butylated hydroxytoluene (BHT), chloroform, ethanol and hexane were purchased form Kanto Chemical (Tokyo, Japan). 1-(3-Dimethylaminopropyl)-3-ethylcarbodiimide hydrochloride (EDC) was purchased from Tokyo Chemical Industry (Tokyo, Japan). All solvents for chemical reaction and reagents used were analytical reagent grade. All LC/ESI-MS/MS analysis solvents (water, methanol, acetonitrile) were used LC/ESI-MS/MS grade and purchased from Kanto Chemical. The Glufatase kit was purchased from Nippon Bio-Test Laboratories (Wako, Japan). Sulfatase from *Helix pomatia* (Type H-2) was purchased

from Sigma-Aldrich.

Preparation of standard solutions

Individual stock solutions of oxysterols were prepared separately at 1 mg/mL in methanol and stored at -25ºC. These solutions were mixed equally for analysis of unknown samples. Five point-calibration standard solutions (0.1, 0.3, 1.0, 3.0, and 10.0 pmol/mL) for LC/ESI-MS/MS analysis were prepared in acetonitrile. Calibration standard solutions were stable in analytical glass tubes for at least 2 weeks at -25ºC.

LC/ESI-MS/MS analysis

The LC/ESI-MS/MS system included a TSQ Quantum Discovery Max mass spectrometer (Thermo Fisher Scientific, San Jose, CA, US) equipped with an ESI probe and Surveyor HPLC system (Thermo Fisher Scientific). A chromatography separation column, InertSustain C8 HP (150 mm × 2.1 mm ID, 3 µm particle size; GL Sciences, Tokyo, Japan), was maintained at 35°C. A mixture of 10 mM ammonium acetate and acetonitrile was used as the eluent, and separation was carried out by linear gradient elution at a flow rate of 0.25 mL/min. Composition of the mobile phase was gradually changed as follows: ammonium acetate-acetonitrile (18:82, v/v) for 2 min, ammonium acetate-acetonitrile (14:86, v/v) for 2-33 min, ammonium acetate-acetonitrile (2:98, v/v) for 33-34 min, and ammonium acetate-acetonitrile

(2:98, v/v) for 4 min. While operating the LC/ESI-MS/MS apparatus, the spray voltage and vaporizer temperature were set at 3000 V and 330°C, respectively. The sheath and auxiliary gas (nitrogen) pressures were set at 60 and 12 arbitrary units, respectively, while the ion transfer capillary temperature was 330°C. The collision gas (argon) pressure and the collision energy were kept at 1.3 mm Torr and 7-39 eV respectively, all in the positive ion mode.

Supplementary Figure

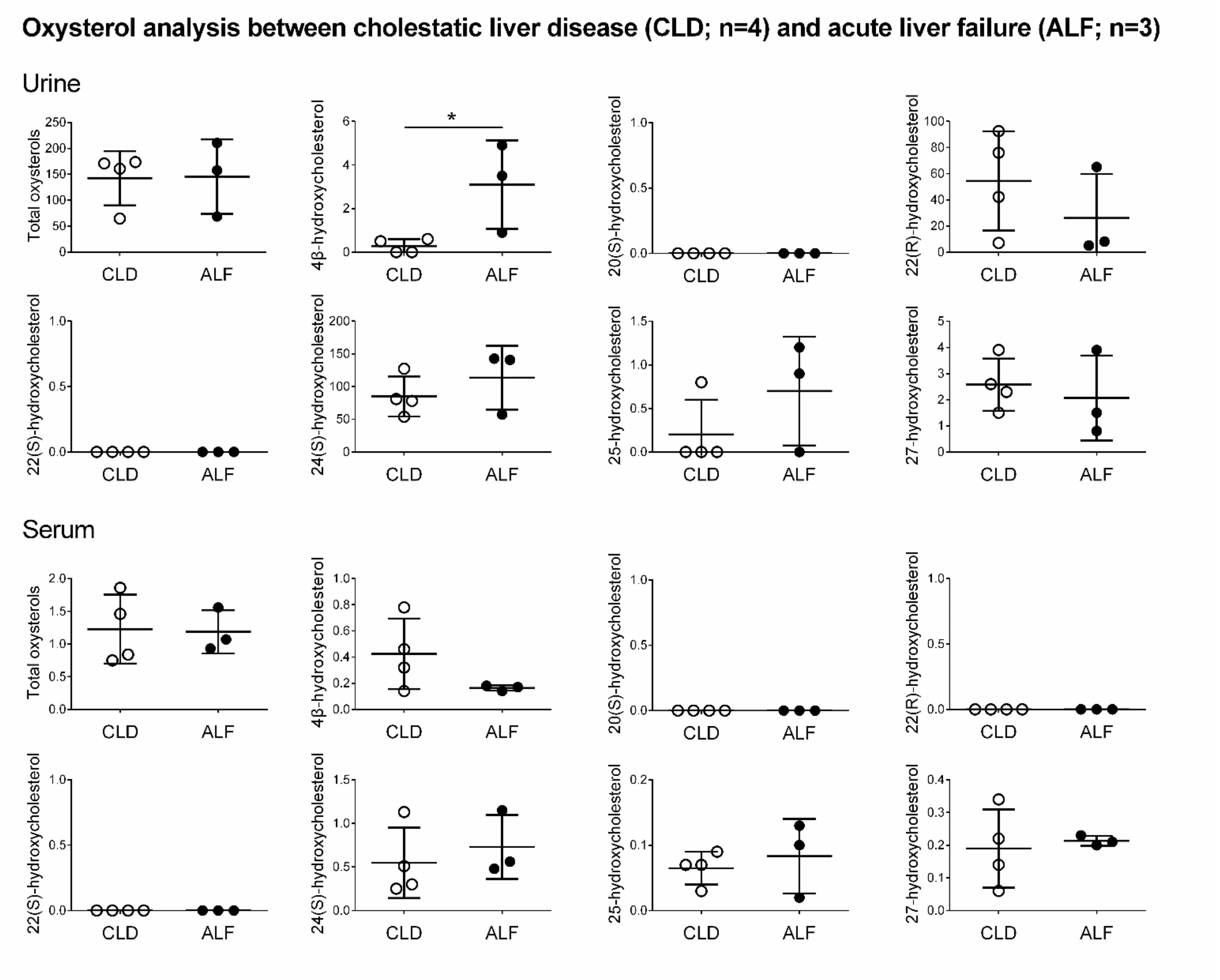

Supplement: Supplementary file 1 — Supplementary Information. [file 41598_2020_63758_MOESM1_ESM.pdf]
